# The Chinese version of the Functionality Appreciation Scale: Psychometric properties and measurement invariance across gender and age

**DOI:** 10.1186/s40337-023-00826-8

**Published:** 2023-06-20

**Authors:** Jinbo He, Tianxiang Cui, Wesley R. Barnhart, Gui Chen

**Affiliations:** 1 School of Humanities and Social Science, The Chinese University of Hong Kong, Shenzhen, Shenzhen, 518172 China; 2 Department of Psychology, University of Macau, Macau, China; 3 Department of Psychology, Bowling Green State University, Bowling Green, OH USA; 4 College of Educational Science, Hengyang Normal University, Hengyang, China

**Keywords:** Body image, Functionality appreciation, Validation, Chinese, Invariance

## Abstract

**Background:**

Functionality appreciation, as an important aspect of positive image, is associated with fewer body image disturbances, fewer disordered eating behaviors, and improved psychological well-being. However, it has been under-researched in Asian countries. The current work aimed to examine the psychometric properties of the Functionality Appreciation Scale (FAS) among four Chinese samples of different ages, and further examine measurement invariance and differences of the FAS across gender and age groups.

**Methods:**

Exploratory and confirmatory factor analyses (EFA and CFA) were conducted to examine the factorial structure of the FAS among four Chinese samples of different ages, including middle school adolescents (*n* = 894, *M*_*age*_ = 12.17 years), high school adolescents (*n* = 1347, *M*_*age*_ = 15.07 years), young adults (*n* = 473, *M*_*age*_ = 21.95 years), and older adults (*n* = 313, *M*_*age*_ = 67.90 years). The measurement invariance of the FAS across gender and age was examined. Internal consistency reliability and construct validity were evaluated.

**Results:**

The FAS had a unidimensional structure and was invariant across gender and age groups. The FAS presented sound psychometric properties in all age groups by gender, with good internal consistency reliability [e.g., high Cronbach’s *α* values (.91 ~ .97)] and good construct validity (e.g., significant associations with body appreciation, body dissatisfaction, and disordered eating). Moreover, group comparisons showed minimal gender differences in functionality appreciation. However, significant age differences were found in functionality appreciation, with older ages generally associated with higher functionality appreciation.

**Conclusion:**

Overall, findings suggest that the FAS is a sound instrument to be used in the Chinese context. Furthermore, functionality appreciation was found to be higher in older adults than adolescents or young adults, suggesting the potential important role of aging in functionality appreciation.

## Abstract (plain language summary)

Functionality appreciation, as a positive body image component emphasizing the appreciation on one’s body for what it can do, is associated with fewer body image disturbances, fewer disordered eating behaviors, and improved psychological well-being. The Functionality Appreciation Scale (FAS) measures functionality appreciation and has been widely validated in a number of different populations. However, the psychometric properties of the FAS have not been systematically examined in the Chinese context, especially for adolescents and older adults. Results indicated that the psychometric properties of the FAS were sound and could be equally used to assess functionality appreciation in Chinese adolescents, young adults, and older adults.

## Background

Positive body image is a multifaceted construct encompassing an overall love, respect, and acceptance of one’s body [1]. As an important but under-researched aspect of positive body image, functionality appreciation refers to *appreciating what the body can do or is capable of doing* [2]. Functionality appreciation has been found to have consistent negative relations with negative body image (e.g., body dissatisfaction and body surveillance) [3–5] and eating disorder symptomatology [2, 5–7]. Furthermore, functionality appreciation is positively related to body appreciation [2, 3], body image flexibility [8], adaptive eating behaviors (e.g., intuitive eating) [2, 6], and measures of well-being (e.g., life satisfaction and self-esteem [5, 7, 9, 10]. Experimental data have been promising in positioning body functionality as a way to improve body image, reduce self-objectification, and potentially temper negative consequences associated with exposure to body-ideal images in the media [11–14]. Thus, interventions for improving functionality appreciation have been suggested to be a potential avenue for preventing disordered eating behaviors [5, 15].

To measure functionality appreciation, Alleva et al. [2] developed the Functionality Appreciation Scale (FAS) which contains 7 items and has a unidimensional structure. The FAS has been validated in many Western adult samples and showed sound psychometric properties. Specifically, in Alleva et al. [2], the FAS showed excellent internal consistency reliability, good construct validity, and measurement invariance by gender. After the development of the FAS, the FAS was validated and presented good psychometric properties in both adolescent and adult samples from various countries, such as adolescent populations in Iran [16] and adult populations in Australia [8], Italy [17], Japan [18], Malay [9], and Romania [7]. According to a recent meta-analytic study that included studies using the FAS to explore the relationship between functionality appreciation and its correlates [5], functionality appreciation is consistently associated with fewer body image disturbances, fewer disordered eating behaviors, and improved psychological well-being. Specifically, in a prospective study, higher baseline functionality appreciation predicted a lower likelihood of the onset of seven key symptoms of eating disorders (e.g., binge eating, purging, and fasting) at an 8-month follow-up [15].

Even though there has been some preliminary research on functionality appreciation in Asian countries, the majority of research on functionality appreciation with the FAS has been undertaken in Western, Educated, Industrialized, Rich, and Democratic (WEIRD [19]) countries [16]. This discrepancy in the research on functionality appreciation is important given China has a different cultural context compared to WEIRD countries. Moreover, Chinese people account for about 18% of the world’s population [20] and China also has a long and unique history of body self-caring (called “yangsheng”) [21] that is different from Western and other Asian countries. Indeed, China and Western societies stress different cultural values (i.e., collectivism vs individualism, interdependence vs independence [22]) which have been found to affect individuals’ body image (e.g., body dissatisfaction) [23, 24]. For instance, collectivistic cultural values of relatedness, social harmony, and blending in [25] may increase Chinese individuals’ societal pressures to conform to societal body ideals (e.g., thinness for women [26]) to blend in such that Chinese individuals may have greater focus on their physical appearance than their functionality. Thus, it is possible that Chinese people may hold different opinions on functionality appreciation from Western people. Although the Chinese version of the FAS (C-FAS) has been available and used in Chinese young adults [27, 28] and older adults [10], its psychometric properties have not been fully examined, especially in Chinese adolescents and older adults. Thus, more psychometric research is warranted on functionality appreciation in Chinese populations. Moreover, body image disturbances and disordered eating/eating disorders are prevalent in China across different age groups, including both adolescents and adults [29–31]. There is also accumulating empirical evidence suggesting that body image disturbances and eating disorders occur in older adults [32, 33]. Thus, it is of importance to test the psychometric properties of the FAS across the lifespan for the future design and refinement of functionality appreciation interventions to prevent body image disturbances and disordered eating/eating disorders in Chinese individuals across the lifespan.

In addition to this gap in the literature, there are limited studies worldwide that have considered age differences when measuring functionality appreciation. Importantly, body image constructs such as functionality appreciation bear relevance throughout one’s life span [34], and individuals at different life stages may experience functionality appreciation differently. Put another way, aspects of one’s body image are associated with age [35–38], and thus it should be of interest to further explore age differences in functionality appreciation. However, to our knowledge, even though the FAS has been validated in adolescents and adults, no studies have explored whether the FAS is invariant across different age groups (e.g., adolescents vs. adults). In addition, the FAS has been found to be measurement invariant across gender in previous validation studies among adults, and studies generally reported no gender differences in scores on the FAS [2, 7–9, 17]. But it remains unclear whether there are gender differences in the Chinese context due to the limited research evidence on functionality appreciation in China. Nevertheless, according to previous studies reporting gender differences in self-objectification (i.e., the act of individuals focusing more on physical appearance than on what their body can do [39]) in Chinese samples, Chinese young women had generally higher self-objectification than Chinese young men [40–42]. Given the close link between functionality appreciation and self-objectification [13], it is of interest to explore whether gender differences in functionality appreciation exist in the Chinese context, which may help explain the well-documented gender differences in self-objectification in Chinese individuals. In addition, whether there are gender differences in different age groups, especially in adolescents and older adults, remains unknown in the Chinese context.

## The current study

In light of the discussions above, more research is warranted on the psychometric properties of the FAS in China, including differences in functionality appreciation by gender and across the lifespan. Therefore, the current study aimed to examine the psychometric properties of FAS among Chinese adolescents, young adults, and older adults. We also further tested whether the C-FAS was invariant across gender and age and examined gender and age differences in the C-FAS scores. More specifically, Study 1 examined the psychometric properties and measurement invariance by gender for the C-FAS among adolescents; Study 2 examined the psychometric properties and measurement invariance across gender for the C-FAS among young adults; Study 3 examined the psychometric properties and measurement invariance by gender for the C-FAS among older adults; and Study 4 examined measurement invariance across the four age groups for the C-FAS. Across the four studies, we hypothesized that: (1) the C-FAS would present a unidimensional factor structure; (2) the C-FAS would have an adequate internal consistency reliability; (3) The C-FAS would be invariant across gender and age, and there would be no gender differences and age differences in the scores of the C-FAS; and (4) the scores of C-FAS would demonstrate construct validity via positive associations with positive psychological measures (e.g., body appreciation) and negative associations with negative psychological measures (e.g., eating disorder symptomatology).

## Study 1

Study 1 examined the psychometric properties and measurement invariance by gender of the FAS in Chinese adolescents.

### Method

#### Participants and procedure

The protocol of this study as a large-scale, longitudinal survey was approved by the research ethics office of Hengyang Normal University. More details about the project can be found in our previous publication using baseline data [43]. One middle school and one high school from Hunan Province were involved in data collection. Psychology teachers, who were responsible for the weekly psychological health classes for the students in the two schools, introduced the project to the students during class. If students agreed to participate, they were asked to bring a consent form home for their parents' or other custodians’ (e.g., grandparents’) further agreement. To ensure participants provided quality responses, we added two attention-check questions (e.g., “please select ‘strongly agree’ for this item”). Finally, at baseline, 2713 middle and high school students and their parents agreed to participate in the project by providing informed consent. After removing the incomplete surveys (e.g., those failing to complete 50% of the questionnaires in the survey) and invalid surveys that failed to pass the attention check questions, 894 middle school adolescents (47.3% boys) were included in the current study, with a mean (*SD*) age of 12.17 (0.50) years and a mean (*SD*) BMI of 18.26 (2.98) kg/m^2^. Regarding high school adolescents, 1347 (45.7% boys) were included, with a mean (*SD*) age of 15.07 (0.57) years and a mean (*SD*) BMI of 19.47 (2.64) kg/m^2^.

#### Measures

*Functionality appreciation* The FAS has 7 items which are rated on a 5-point Likert scale ranging from 1 (*strongly disagree*) to 5 (*strongly agree*) (Alleva et al. [2]. Higher average scores of the 7 items indicate higher levels of functionality appreciation. The C-FAS showed a one-dimensional model with an adequate fit and good internal consistency reliability in Chinese college women [27]. Even though the C-FAS has been used in adults [10, 27, 28, 44], to ensure the suitability of using it in adolescent samples, the C-FAS was administered to 10 high school students (50% boys) aged from 12 ~ 16 years old. Based on their feedback, all adolescents could correctly interpret each item of the scale. As such, the C-FAS was used in the adolescent sample without any wording modifications.

*Body appreciation* The Body Appreciation Scale-2 (BAS-2) [1] was used to assess body appreciation. The BAS-2 has 10 items (e.g., “I respect my body”) which are rated on a 5-point Likert scale, ranging from 1 (*never*) to 5 (*always*). Higher average scores of the 10 items reflect higher levels of body appreciation. Good psychometric properties (e.g., strong internal consistency reliability) of the BAS-2 were revealed in Chinese samples (e.g., Swami et al. [71]). In the current study, it had a Cronbach’s $\alpha$ of 0.89 and 0.88 in middle and high school students, respectively.

*Body dissatisfaction* The 9-item Body Dissatisfaction subscale of the Eating Disorder Inventory (EDI-BD) [89] was used to measure body dissatisfaction. The items are rated on a 6-point Likert scale ranging from 1 (*never*) to 6 (*always*). Higher total scores represent higher levels of body dissatisfaction. Good psychometric properties (e.g., strong internal consistency reliability and construct validity) of the EDI were found in Chinese samples [45–47]. In the current study, the EDI-BD had a Cronbach’s $\alpha$ of 0.90 and 0.91 in middle and high school students, respectively.

*Body image inflexibility* The 5-item Body Image-Acceptance and Action Questionnaire (BI-AAQ-5) [48] was used to assess body image inflexibility. The items were rated on a 7-point Likert scale ranging from 1 (*never true*) to 7 (*always true*). Higher average scores indicate higher levels of body image inflexibility. The BI-AAQ-5 exhibited good internal consistency reliability, test–retest reliability, and convergent validity in Chinese young adults [49]. In the current study, it had a Cronbach’s $\alpha$ of 0.89 for both middle and high school students.

*Psychological distress* The 6-item Kessler Psychological Distress Scale (K6) [50] was used to assess psychological distress. The items were rated on a 5-point Likert scale from 0 (*none of the time*) to 4 (*all of the time*). Higher total scores indicate higher levels of psychological distress. The K6 was validated with good reliability and validity in Chinese samples [51]. In the current study, it had a Cronbach’s $\alpha$ of 0.85 and 0.83 in middle and high school students, respectively.

*Eating disorder symptomatology* The 12-item Eating Disorder Examination-Questionnaire (EDE-QS) [52] was used to examine eating disorder symptomatology. The items are rated on a 4-point Likert scale from 0 (*0 days/not at all*) to 3 (*6–7 days/markedly*). Higher total scores indicate higher levels of eating disorder symptomatology. The EDE-QS showed strong internal consistency reliability, test–retest reliability, and convergent validity in Chinese samples [53]. In the current study, the EDE-QS had a Cronbach’s $\alpha$ of 0.86 and 0.85 in middle and high school students, respectively.

*Self-compassion* The 12-item Self-Compassion Scale—Short Form (SCS-SF) [54] was used to measure self-compassion. Response options range from 1 (*almost never*) to 5 (*almost always*). Higher average scores reflect higher levels of self-compassion. The SCS-SF showed adequate internal consistency reliability, test–retest reliability, and construct validity in Chinese samples [55]. In the current study, the SCS-SF had a Cronbach’s $\alpha$ of 0.71 and 0.73 in middle and high school students, respectively.

*Body Mass Index* BMI was obtained with self-reported height and weight.

#### Statistical analysis

R 4.2.0 [56] was used to conduct data analysis. We adopted the EFA (exploratory factor analysis)-to-CFA (confirmatory factor analysis) approach [57] to test the factor structure of the C-FAS. All EFA and CFA were conducted separately for boys and girls. Thus, of the 894 middle school students, 449 (210 boys and 239 girls) and 445 (213 boys and 232 girls) students were randomly assigned for EFA and CFA, respectively. Of the 1347 high school students, 677 (308 boys and 369 girls) and 670 (308 boys and 362 girls) students were randomly assigned for EFA and CFA, respectively.

Specifically, EFA was conducted via the *psych* package version 2.2.5 [58] with principal-axis factoring, quartimax rotation, and ordinary least squares estimator [59]. In addition, considering the FAS adopted an ordinal Likert-type scale, we used polychoric correlations in EFA [60]. Moreover, to determine the number of factors, we used the parallel analysis as well as the sizes of eigenvalues. Factor loadings higher than 0.4 were considered adequate [61].

Confirmatory factor analysis (CFA) was carried out via the *lavaan* package version 0.6–11 [62]. Considering again that the items of C-FAS are rated on a Likert scale which produces ordinal responses, we used the mean- and variance-adjusted weighted least squares estimator (WLSMV) for model estimation as the WLSMV was found superior to other estimators (e.g., MLR) for Likert-type rating scales [63]. To evaluate model fit, the following model fit indictors were recommended to report [64]: Comparative Fit Index (CFI; ≥ 0.95 indicates a good fit, ≥ 0.90 indicates an acceptable fit), Tucker–Lewis Index (TLI; ≥ 0.95 indicates a good fit, ≥ 0.90 indicates an acceptable fit), Root Mean Square Error of Approximation (RMSEA; ≤ 0.05 indicates a good fit; ≤ 0.08 indicates an acceptable fit), and Standardized Root Mean square Residual (SRMR; ≤ 0.06 indicates a good fit; ≤ 0.08 indicates an acceptable fit). However, it should be noted that the RMSEA was not reported in the current study as it can be unreliable when using ordinal responses by consistently rejecting well-fitted models when sample sizes are large and data contained 5 responses. Instead, SRMR was recommended to be used [65].

Measurement invariance was examined by using the multi-group CFA method which assesses measurement invariance at the configural, metric, and scalar models sequentially. In the configural invariance test, the factor structure was constrained to be equal; in the metric invariance test, factor loadings were constrained to be equal while intercepts were freely estimated; and in the metric invariance test, both factor loadings and intercepts were constrained to be equal. Based on Cheung and Rensvold [66], CFI < 0.01 and SRMR < 0.03 indicate invariance between two nested models (e.g., configural model vs. metric model, and metric model vs. scalar model).

To assess the reliability of the C-FAS, we also used the *psych* package version 2.2.5 [58]. Specifically, we examined the internal consistency reliability of the C-FAS as indicated by the ordinal Cronbach’s *α* (≥ 0.70 suggests acceptable internal consistency reliability), which is more accurate for Likert-type scales [67]. Finally, we evaluated the construct validity of the C-FAS by exploring the bivariate correlations between the scores of C-FAS and theoretically correlated measures. Based on previous research [2, 7–9], we expected significant and positive associations between the C-FAS scores and positive psychological constructs (e.g., body appreciation) and significant and negative correlations with negative psychological constructs (e.g., body dissatisfaction).

For interpreting effect sizes, we relied on the recommendations from Cohen [68]. Specifically, correlations of 0.10, 0.30, and 0.50 were considered small, medium, and large, respectively. For standardized mean differences, Cohen’s *d* was used, for which values of 0.20, 0.50, and 0.80 were small, medium, and large, respectively.

### Results

#### Preliminary analyses

The rate of missing data for the C-FAS items ranged from 0.4 to 0.8% for the middle school sample and 0.3 to 0.7% for the high school sample. The Little’s Test for MCAR (missing completely at random) was significant for middle (*χ*^*2*^ = 56.58, *p* = 0.021) and high school students (*χ*^*2*^ = 83.35, *p* < 0.001). However, as the missing rates were much lower than 5% [69], these missing data were less likely to impact the results. Therefore, we decided not to impute these missing data in subsequent analyses.

#### Exploratory factor analysis

For the half-sample of boys from middle school (*n* = 210), results suggested that KMO = 0.90 and Bartlett’s test of sphericity, *χ*^*2*^(21) = 856.42 (*p* < 0.001), indicating that the C-FAS items were appropriate for EFA. The results of the EFA revealed only one factor that had eigenvalues greater than 1, and parallel analysis showed one factor from the actual data had an eigenvalue greater than those from the random data (λ_1_ = 4.76 > 0.59, λ_2_ = 0.16 < 0.23). Thus, we decided to retain only one factor in EFA, which explained 68% of the common variance. As shown in Table 1, all 7 items loaded strongly onto the extracted factor (item-factor loadings ≥ 0.69).

**Table 1:** Standardized factor loadings of EFA for the one-dimensional structure of the C-FAS by gender for four age groups: Studies 1, 2, and 3

Items	Study 1: Middle school students		Study 1: High school students		Study 2: Young adults (*n* = 473)		Study 3: Older adults (*n* = 313)	
	Boys (*n* = 210)	Girls (*n* = 239)	Boys (*n* = 308)	Girls (*n* = 369)	Men (*n* = 124)	Women (*n* = 113)	Men (*n* = 75)	Women (*n* = 81)
1. I appreciate my body for what it is capable of doing	0.75	0.73	0.80	0.76	0.75	0.75	0.82	0.87
2. I am grateful for the health of my body, even if it isn’t always as healthy as I would like it to be	0.69	0.68	0.71	0.78	0.80	0.68	0.87	0.94
3. I appreciate that my body allows me to communicate and interact with others	0.86	0.92	0.87	0.88	0.80	0.77	0.93	0.95
4. I acknowledge and appreciate when my body feels good and/or relaxed	0.86	0.84	0.84	0.81	0.84	0.70	0.90	0.93
5. I am grateful that my body enables me to engage in activities that I enjoy or find important	0.88	0.89	0.89	0.83	0.74	0.85	0.84	0.95
6. I feel that my body does so much for me	0.84	0.90	0.84	0.84	0.79	0.86	0.84	0.95
7. I respect my body for the functions it performs	0.87	0.87	0.81	0.87	0.87	0.74	0.85	0.93

For the half-sample of girls from middle school (*n* = 211), results suggested that KMO = 0.93 and Bartlett’s test of sphericity, *χ*^*2*^(21) = 1126.00 (*p* < 0.001), indicating that the C-FAS items were appropriate for EFA. The results of the EFA revealed only one factor that had eigenvalues greater than 1, and parallel analysis confirmed the one-factor solution, with only one factor from the actual data having eigenvalues greater than those from the random data (λ_1_ = 4.91 > 0.50, λ_2_ = 0.16 < 0.19). Thus, we adopted the one-factor solution, which explained 70% of the common variance. As shown in Table 1, all 7 items loaded strongly onto the extracted factor (item-factor loadings ≥ 0.68).

For the half-sample of boys from high school (*n* = 308), results suggested that KMO = 0.89 and Bartlett’s test of sphericity, *χ*^*2*^(21) = 1333.88 (*p* < 0.001), indicating that the C-FAS items were appropriate for EFA. Even though EFA showed only one factor that had an eigenvalue greater than 1, parallel analysis indicated two factors (λ_1_ = 4.75 > 0.48, λ_2_ = 0.29 > 0.18, λ_3_ = 0.06 < 0.10). Thus, we proceeded with the two-factor solution. However, the EFA results of the two-factor solution showed that all items strongly loaded on the first factor (i.e., all loadings > 0.40), but no items strongly loaded on the second factor (i.e., all loadings < 0.40). Thus, we decided to retain only one factor in EFA, which explained 68% of the common variance. As shown in Table 1, all 7 items loaded strongly onto the extracted factor (item-factor loadings ≥ 0.71).

For the half-sample of girls from high school (*n* = 369), results suggested that KMO = 0.90 and Bartlett’s test of sphericity, *χ*^*2*^(21) = 1571.58 (*p* < 0.001), indicating that the C-FAS items were appropriate for EFA. The results of EFA were similar to those of high school boys. Specifically, one factor had an eigenvalue greater than 1, but parallel analysis suggested two factors (λ_1_ = 4.76 > 0.42, λ_2_ = 0.25 > 0.16, λ_3_ = 0.08 < 0.09). However, no items were strongly loaded on the second factor. Thus, we adopted the one-factor solution, which explained 68% of the common variance. As shown in Table 1, all 7 items loaded strongly onto the extracted factor (item-factor loadings ≥ 0.76).

#### Confirmatory factor analysis

For both middle and high school boys and girls, the fit indices provided adequate support for a unidimensional model of the C-FAS, with *χ*^*2*^ = 203.92 (*df* = 14, *p* < 0.001), CFI = 0.95, TLI = 0.92, and SRMR = 0.06 for middle school boys; *χ*^*2*^ = 141.58 (*df* = 14, *p* < 0.001), CFI = 0.97, TLI = 0.96, and SRMR = 0.04 for middle school girls; *χ*^*2*^ = 181.95 (*df* = 14, *p* < 0.001), CFI = 0.98, TLI = 0.96, and SRMR = 0.05 for high school boys; and *χ*^*2*^ = 278.50 (*df* = 14, *p* < 0.001), CFI = 0.97, TLI = 0.95, and SRMR = 0.06 for high school girls. For the boys and girls from both samples, the standardized estimates of loadings ranged from 0.70 to 0.91 (see Table 2).

**Table 2:** Standardized factor loadings of CFA for the one-dimensional structure of the C-FAS by gender four age groups: Studies 1, 2, and 3

Items	Study 1: Middle school students		Study 1: High school students		Study 2: Young adults		Study 3: Older adults	
	Boys (*n* = 213)	Girls (*n* = 232)	Boys (*n* = 308)	Girls (*n* = 362)	Men (*n* = 122)	Women (*n* = 114)	Men (*n* = 76)	Women (*n* = 81)
1. I appreciate my body for what it is capable of doing	0.75	0.72	0.71	0.80	0.72	0.78	0.88	0.89
2. I am grateful for the health of my body, even if it isn’t always as healthy as I would like it to be	0.74	0.77	0.73	0.70	0.65	0.65	0.90	0.92
3. I appreciate that my body allows me to communicate and interact with others	0.86	0.88	0.87	0.88	0.84	0.83	0.92	0.87
4. I acknowledge and appreciate when my body feels good and/or relaxed	0.89	0.89	0.82	0.90	0.87	0.79	0.96	0.93
5. I am grateful that my body enables me to engage in activities that I enjoy or find important	0.84	0.87	0.89	0.88	0.90	0.86	0.95	0.88
6. I feel that my body does so much for me	0.90	0.92	0.91	0.86	0.71	0.84	0.97	0.96
7. I respect my body for the functions it performs	0.87	0.84	0.91	0.90	0.62	0.83	0.92	0.92

#### Gender invariance and reliability

Next, we examined the measurement invariance of the one-dimensional model of the C-FAS scores across gender. As described in Table 3, all indices suggested that configural, metric, and scalar invariance were supported across gender for both middle and high school samples. In line with Swami et al. [7], we further conducted independent-samples *t*-tests. Results showed that there were no gender differences in the C-FAS scores for either middle school [(boys, *M* = 3.82, *SD* = 0.88; girls, *M* = 3.77, *SD* = 0.84), *t* (889) = 0.86, *p* = 0.390, *d* = 0.06] or high school [(boys, *M* = 3.91, *SD* = 0.71; girls, *M* = 3.94, *SD* = 0.67), *t* (1275.41) = -0.83, *p* = 0.408, *d* = -0.04] students.

**Table 3:** Measurement invariance tests across gender for the four age groups

	${\chi }^{2}$	*df*	CFI	TLI	SRMR	$\Delta$CFI	$\Delta$SRMR
*Middle school students* (*n* = 894)							
Configural model	401.102***	28	0.974	0.961	0.035		
Metric model	285.383***	34	0.982	0.978	0.037	0.008	0.002
Scalar model	308.267***	54	0.982	0.986	0.035	0.000	− 0.002
*High school students* (*n* = 1347)							
Configural model	875.542***	28	0.963	0.944	0.050		
Metric model	689.251***	34	0.971	0.965	0.051	0.008	0.001
Scalar model	706.919***	54	0.971	0.978	0.050	0.000	− 0.001
*Young adults *(*n* = 473)							
Configural model	148.970***	28	0.976	0.964	0.042		
Metric model	151.466***	34	0.976	0.971	0.051	0.000	0.009
Scalar model	147.818***	54	0.981	0.985	0.044	0.005	− 0.007
*Older adults* (*n* = 313)							
Configural model	174.514***	28	0.990	0.984	0.035		
Metric model	146.455***	34	0.992	0.990	0.036	0.002	0.001
Scalar model	158.614***	54	0.993	0.994	0.035	− 0.001	− 0.001

***p < .001

Regarding the reliability of the C-FAS, results showed that the ordinal Cronbach’s $\alpha$ for the C-FAS was 0.94 for middle school boys and girls as well as high school boys and girls, suggesting good internal consistency reliability of the scale in Chinese adolescents.

#### Construct validity

As shown in Table 4, For both middle and high school boys, functionality appreciation was significantly and positively correlated with body appreciation (medium to large effect sizes) and self-compassion (small to medium effect sizes), and negatively correlated with body dissatisfaction (small to medium effect sizes), body image inflexibility (small to medium effect sizes), psychological distress (small to medium effect sizes), and eating disorder symptomatology (small to medium effect sizes). For both middle and high school girls, functionality appreciation was also significantly and positively correlated with body appreciation (medium to large effect sizes) and self-compassion (small to medium effect sizes), and negatively correlated with body dissatisfaction (small to medium effect sizes), body image inflexibility (small effect sizes), psychological distress (small to medium effect sizes), and eating disorder symptomatology (small to medium effect sizes). In addition, for all sub-samples (i.e., middle school boys, middle school girls, high school boys, and high school girls), the correlations between functionality appreciation and BMI were small and nonsignificant.

**Table 4:** Bivariate correlations between functionality appreciation and other constructs for Study 1 (adolescents)

	1	2	3	4	5	6	7	8
*Middle school students* (*n* = 894)								
1. Functionality appreciation		.40***	− .26***	− .12**	− .17***	− .14**	.24***	− .06
2. Body appreciation	.34***		− .49***	− .39***	− .43***	− .43***	.50***	− .15**
3. Body dissatisfaction	− .16**	− .40***		.55***	.39***	.64***	− .43***	.50***
4. Body image inflexibility	− .09	− .23***	.49***		.52***	.70***	− .43***	.28***
5. Psychological distress	− .17***	− .31***	.24***	.42***		.49***	− .58***	.10
6. Eating disturbances	− .11*	− .23***	.58***	.71***	.45***		− .40***	.38***
7. Self− compassion	.23***	.37***	− .33***	− .32***	− .43***	− .22***		− .05
8. BMI	− .03	− .07	.45***	.28***	.07	.39***	− .04	
Boys								
M	3.82	3.92	26.89	10.93	5.43	6.30	3.29	18.60
SD	0.88	0.75	11.23	6.23	4.34	6.59	0.57	3.29
Girls								
M	3.77	3.89	30.61	11.31	6.05	6.84	3.20	17.96
SD	0.84	0.83	10.71	6.59	4.85	6.56	0.63	2.65
*High school students* (*n* = 1347)								
1. Functionality appreciation		.40***	− .20***	− .11**	− .17***	− .07	.30***	− .04
2. Body appreciation	.46***		− .46***	− .39***	− .41***	− .38***	.49***	− .17***
3. Body dissatisfaction	− .26***	− .43***		.57***	.31***	.59***	− .34***	.51***
4. Body image inflexibility	− .11**	− .28***	.49***		.41***	.74***	− .38***	.30***
5. Psychological distress	− .11**	− .33***	.18***	.31***		.40***	− .57***	.08
6. Eating disturbances	− .10*	− .24***	.51***	.72***	.31***		− .31***	.33***
7. Self-compassion	.24***	.36***	− .23***	− .24***	− .43***	− .20***		− .01
8. BMI	.01	− .05	.51***	.39***	− .03	.43***	.04	
Boys								
M	3.91	3.68	28.27	9.66	6.98	4.86	3.10	19.72
SD	0.71	0.72	10.03	5.19	4.24	5.02	0.51	3.02
Girls								
M	3.94	3.62	35.08	11.80	8.03	7.54	3.04	19.27
SD	0.67	0.74	10.15	6.13	4.29	6.30	0.51	2.26

For both middle and high school students’ correlation matrices, girls’ correlations are on the top diagonals and boys’ correlations are on the bottom diagonals

*p < .05, **p < .01, ***p < .001

## Study 2

Study 2 examined the psychometric properties and measurement invariance across gender of the C-FAS in Chinese young adults.

### Method

#### Participants and procedure

The protocol of this study was approved by the Institutional Review Board (IRB) of The Chinese University of Hong Kong, Shenzhen. By using an online survey platform, Credamo, 480 young adults (18–25 years old) [70] were surveyed. After completion of the survey, participants would receive 10 ¥ ($1.41) as compensation. By removing those who failed to correctly answer the two attention check questions (e.g., “please select BANANA as an answer so that we know you are paying attention while doing the survey.”), 473 were left. Of them, 246 (52.0%) were men and 227 (48.0%) were women. They had a mean (*SD*) age of 21.95 (2.27) years and a mean (*SD*) BMI of 20.23 (3.18) kg/m^2^.

#### Measures

Except for the SCS-SF,^1^ the measures used in Study 1 were also used in Study 2. For other measures (see Study 1 for descriptions of these measures), the Cronbach’s $\alpha$ was 0.90 for the BAS-2 [71], 0.88 for the EDI-BD [45], 0.86 for the BI-AAQ-5 [49], 0.85 for the K6 [51], and 0.88 for the EDE-QS [53]. BMI was also obtained with self-reported height and weight.

#### Statistical analysis

We used the same statistical software (i.e., R 4.2.0), statistical packages (i.e., *lavan* and *psych*), and statistical methods (e.g., EFA and CFA) used in Study 1. In addition, of the 473 young adults included in Study 2, 237 (124 men and 113 women) and 236 (122 men and 114 women) were assigned randomly to EFA and CFA, respectively.

### Results

#### Exploratory factor analysis

For the half-sample of young adult men (*n* = 124), results suggested that KMO = 0.88 and Bartlett’s test of sphericity, *χ*^*2*^(21) = 393.79 (*p* < 0.001), indicating that the C-FAS items were appropriate for EFA. The results of the EFA showed one factor that had an eigenvalue greater than 1, and parallel analysis confirmed the retainment of one factor (λ_1_ = 4.47 > 0.84, λ_2_ = 0.20 < 0.30). The one-factor solution could explain 64% of the common variance. As shown in Table 1, all 7 items loaded strongly onto the extracted factor (item-factor loadings ≥ 0.74).

For the half-sample of young adult women (*n* = 113), results suggested that KMO = 0.86 and Bartlett’s test of sphericity, *χ*^*2*^(21) = 308.06 (*p* < 0.001), indicating that the C-FAS items were appropriate for EFA. The results of the EFA showed one factor that had an eigenvalue greater than 1, and parallel analysis confirmed the retainment of one factor (λ_1_ = 4.11 > 0.78, λ_2_ = 0.23 < 0.30). The one-factor solution could explain 59% of the common variance. As shown in Table 1, all 7 items loaded strongly onto the extracted factor (item-factor loadings ≥ 0.68).

#### Confirmatory factor analysis

The fit indices provided adequate support for a unidimensional model of the C-FAS for young adults, with *χ*^*2*^ = 53.08 (*df* = 14, *p* < 0.001), CFI = 0.97, TLI = 0.95, and SRMR = 0.06 for young adult men, and with *χ*^*2*^ = 27.50 (*df* = 14, *p* = 0.017), CFI = 0.99, TLI = 0.99, and SRMR = 0.04 for young adult women. The standardized estimates of loadings ranged from 0.62 to 0.90 (see Table 2).

#### Gender invariance and reliability

The measurement invariance test of the one-factor structure of the C-FAS across gender suggested configural, metric, and scalar invariance across gender (see Table 3). An independent-sample *t*-test showed that there was no gender differences in the C-FAS scores (men, *M* = 4.04, *SD* = 0.63 and women, *M* = 4.13, *SD* = 0.57; *t* (471) = -1.57, *p* = 0.116, *d* = 0.15; small effect size). The ordinal Cronbach’s $\alpha$ for the C-FAS in the total sample was 0.91 for both young men and young women, suggesting good internal consistency reliability of the scale in Chinese young adults.

#### Construct validity

As shown in Table 5, for both men and women, functionality appreciation was significantly and positively correlated with body appreciation (medium to large effect sizes), and significantly and negatively correlated with body dissatisfaction (small to medium effect sizes), psychological distress (small to medium effect sizes), and eating disorder symptomatology (small to medium effect sizes). In addition, for both men and women, the correlations between functionality appreciation and BMI and between functionality appreciation and body image inflexibility were very small and nonsignificant.

**Table 5:** Bivariate correlations between functionality appreciation and other constructs for Study 2 (young adults)

Young adults (*n* = 473)	1	2	3	4	5	6	7
1. Functionality appreciation		.40***	− .16*	− .14	− .31***	− .09	− .07
2. Body appreciation	.53***		− .66***	− .39***	− .42***	− .35***	− .27**
3. Body dissatisfaction	− .30***	− .57***		.36***	.32***	.44***	.49***
4. Body image inflexibility	− .09	− .25***	.32***		.39***	.73***	.26***
5. Psychological distress	− .18**	− .34***	.30***	.46***		.42***	.10
6. Eating disturbances	− .16*	− .24***	.40***	.63***	.48***		.24**
7. BMI	− .05	− .11	.36***	− .10	− .13*	.03	
Men							
M	4.04	3.72	27.56	15.56	6.33	8.79	20.86
SD	0.63	0.68	9.30	6.01	4.07	6.78	3.25
Women							
M	4.13	3.55	32.97	14.85	7.61	9.89	19.55
SD	0.57	0.76	10.36	6.96	4.84	7.40	2.97

Women’s correlations are on the top diagonal and men’s correlations are on the bottom diagonal

*p < . 05, **p < . 01, ***p <  .001

## Study 3

Study 3 examined the psychometric properties and measurement invariance by gender of the C-FAS in Chinese older adults.

### Method

#### Participants and procedure

The current study was approved by the IRB of The Chinese University of Hong Kong, Shenzhen. Two trained research assistants recruited potential participants from three cities, namely, Shenzhen, Guangzhou, and Qiqihar by using convenience sampling and snowball sampling (e.g., going to elderly activity centers and asking participants to recommend other potential participants). To recruit older adults, we limited their age to over 50 years old to be consistent with previous literature [72]. Finally, 313 older adults (151 men; 48.2%) were included in the current study. They aged from 51 to 92 years old (*M* = 67.90, *SD* = 7.94). Their BMI ranged from 13.67 to 36.75 kg/m^2^ (*M* = 22.70, *SD* = 3.36). All participants provided written consent prior to participation. All information from these participants was obtained using paper–pencil surveys. Upon completion of the questionnaires, each participant received a gift worth about 10 ¥ ($1.41). Several papers with distinct topics have been published based on the current sample [10, 73, 74].

#### Measures

Except for the SCS-BF,^2^ the measures used in the current sample were the same as in Study 1. In the current sample, the Cronbach’s $\alpha$ was 0.95, 0.95, 0.90, 0.85, and 0.91 for the BAS-2 [71], EDI-BD [45], BI-AAQ-5 [49], K6 [51], and the EDE-QS [53], respectively.

#### Statistical analysis

We used the same statistical software (i.e., R 4.2.0), statistical packages (i.e., *lavan and psych*), and statistical methods used in Studies 1 and 2. In the current study, of the 313 older adults, 156 (75 men and 81 women) and 157 (76 men and 81 women) were assigned randomly for EFA and CFA, respectively.

### Results

#### Exploratory factor analysis

For the half-sample of older adult men (*n* = 75), results suggested that KMO = 0.88 and Bartlett’s test of sphericity, *χ*^*2*^(21) = 343.82 (*p* < 0.001), indicating that the C-FAS items were appropriate for EFA. The results of the EFA showed one factor that had an eigenvalue greater than 1, and parallel analysis confirmed the retainment of one factor (λ_1_ = 5.22 > 1.13, λ_2_ = 0.25 < 0.41). The one-factor solution could explain 75% of the common variance. As shown in Table 1, all 7 items loaded strongly onto the extracted factor (item-factor loadings ≥ 0.82).

For the half-sample of older adult women (*n* = 81), results suggested that KMO = 0.90 and Bartlett’s test of sphericity, *χ*^*2*^(21) = 683.55 (*p* < 0.001), indicating that the C-FAS items were appropriate for EFA. The results of the EFA showed one factor that had an eigenvalue greater than 1, and parallel analysis confirmed the retainment of one factor (λ_1_ = 6.06 > 0.90, λ_2_ = 0.16 < 0.33). The one-factor solution could explain 87% of the common variance. As shown in Table 1, all 7 items loaded strongly onto the extracted factor (item-factor loadings ≥ 0.87).

#### Confirmatory factor analysis

The fit indices provided perfect support for a unidimensional model of the C-FAS with *χ*^*2*^ = 42.55 (*df* = 14, *p* < 0.001), CFI = 0.99, TLI = 0.99, and SRMR = 0.04 for older adult men (*n* = 76), and with *χ*^*2*^ = 48.88 (*df* = 14, *p* < 0.001), CFI = 0.99, TLI = 0.98, and SRMR = 0.04 for older adult women (*n* = 81). For the overall and gender-specific sub-samples, the standardized estimates of loadings ranged from 0.87 to 0.97 (see Table 2).

#### Gender invariance and reliability

Measurement invariance tests showed that the one-factor model of the C-FAS had configural, metric, and scalar invariance across gender, as indicated by the indices (see Table 3). An independent-sample *t*-test showed that there was a significant gender difference in C-FAS scores, with men (*M* = 4.29, *SD* = 0.78) having a higher level of functionality appreciation than women (*M* = 4.10, *SD* = 0.86), *t* (311) = 2.05, *p* = 0.041, *d* = 0.23, a small effect size.

The ordinal Cronbach’s $\alpha$ for the C-FAS in the current sample was 0.97 for both older men and older women, suggesting excellent internal consistency reliability of the scale in Chinese older adults.

#### Construct validity

As shown in Table 6, for both older men and women, functionality appreciation was significantly and positively correlated with body appreciation (large effect sizes), and significantly and negatively correlated with body dissatisfaction (medium effect sizes), body image inflexibility, and eating disorder symptomatology (small to medium effect sizes). Unlike the results in other samples showing nonsignificant (young adults) or significant but small (adolescents) correlations between functionality appreciation and body image inflexibility, for both older men and women, functionality appreciation and body image inflexibility were significantly and negatively related with medium-sized relations (*r* = − 0.37, *p* < 0.001 for both men and women). Interestingly, correlations between functionality appreciation and BMI were negative and significant (men, *r* = − 0.19, *p* = 0.018; women, *r* = − 0.17, *p* = 0.027; small effect sizes).

**Table 6:** Bivariate correlations between functionality appreciation and other constructs for Study 3 (older adults)

Older adults (*n* = 313)	1	2	3	4	5	6	7
1. Functionality appreciation		.55***	− .36***	− .37***	− .24**	− .29***	− .17*
2. Body appreciation	.52***		− .49***	− .48***	− .40***	− .44***	− .28***
3. Body dissatisfaction	− .44***	− .36***		.68***	.26**	.56***	.42***
4. Body image inflexibility	− .37***	− .52***	− .54***		.40***	.61***	.30***
5. Psychological distress	− .09	− .30***	− .23**	.32***		.30***	.18*
6. Eating disturbances	− .22**	− .13	− .25**	.38***	.18*		.44***
7. BMI	− .19*	− .07	.43***	.18*	.03	.16*	
Men							
M	4.29	4.03	22.05	9.82	3.48	3.11	23.13
SD	0.78	0.81	10.52	5.33	3.43	4.68	3.34
Women							
M	4.09	3.91	23.49	10.13	4.14	4.54	22.29
SD	0.85	0.93	12.20	5.69	4.36	6.69	3.33

Women’s correlations are on the top diagonal and men’s correlations are on the bottom diagonal

*p < .05, **p < .01, ***p < .001

## Study 4

Study 4 examined measurement invariance across the four age groups (Chinese middle school students, high school students, young adults, and older adults) for the C-FAS.

### Method

#### Participants and procedure

In the current study, the samples from Studies 1, 2, and 3 were used.

#### Statistical analysis

We used the same statistical software (i.e., R 4.2.0), statistical packages (i.e., *lavaan* and *psych*), and statistical methods (e.g., CFA) used in Studies 1, 2, and 3. In addition, the ratios of unbalanced sample sizes in different groups were within the simulated ratios leading to unbiased metric and scalar invariance tests [75].

### Results

#### Age invariance and mean differences

As described in Table 7, the one-factor model of the C-FAS had configural, metric, and scalar invariance across four age groups. Thus, it is appropriate to make group comparisons by age in functionality appreciation. We used ANOVA tests to examine whether there were age differences by gender in functionality appreciation. The results are described in Table 8. For boys and men, a significant overall difference was found, with *F* (3, 1429) = 15.77 (*p* < 0.001). Follow-up post-hoc tests with Bonferroni corrections (i.e., the corrected $\alpha =0.008$) showed that older adult men (*M* = 4.29, *SD* = 0.78) had the highest levels of functionality appreciation when compared with middle school boys (*M* = 3.82, *SD* = 0.88; *d* = 0.56, *p* < 0.001; a medium effect size), high school boys (*M* = 3.91, *SD* = 0.71; *d* = 0.51, *p* < 0.001; a medium effect size), and young adult men (*M* = 4.04, *SD* = 0.63; *d* = 0.34, *p* = 0.007; a small effect size). Young adult men (*M* = 4.04, *SD* = 0.63) also had higher levels of functionality appreciation than middle school boys (*M* = 3.82, *SD* = 0.88;* d* = 0.29, *p* = 0.001; a small effect size). However, there was no difference between middle school boys (*M* = 3.82, *SD* = 0.88) and high school boys (*M* = 3.91, *SD* = 0.71), with *d* = 0.11, *p* = 0.340, a small effect size.

**Table 7:** Measurement invariance tests by age for males and females for Study 4

	${\chi }^{2}$	*df*	CFI	TLI	SRMR	$\Delta$CFI	$\Delta$SRMR
*Boys and men (n = 1436)*							
Configural model	723.956***	56	0.973	0.959	0.043		
Metric model	558.287***	74	0.980	0.978	0.049	0.007	0.006
Scalar model	630.384***	134	0.980	0.987	0.044	0.000	− 0.005
*Girls and women* (*n* = 1591)							
Configural model	839.965***	56	0.977	0.965	0.042		
Metric model	722.767***	74	0.981	0.978	0.051	0.004	0.009
Scalar model	781.188***	134	0.981	0.988	0.042	0.000	− 0.009

***p < .001

**Table 8:** Age differences in functionality appreciation by gender

	Middle school students (*n* = 894)	High school students (*n* = 1347)	Young adults (*n* = 473)	Older adults (*n* = 313)	*F*	*df*_*between*_	*df*_*within*_
	*Mean* (*SD*)	*Mean* (*SD*)	*Mean* (*SD*)	*Mean* (*SD*)			
Boys and men	3.82 (0.88)^a^	3.91 (0.71)^a,b^	4.04 (0.63)^b^	4.29 (0.78)^d^	15.77***	3	1429
Girls and women	3.77 (0.84)^a^	3.94 (0.67)^b^	4.13 (0.57)^c^	4.10 (0.86)^b,c^	15.70***	3	1585

Superscripts (a, b, c, d) that differ represent significant pairwise differences between the age groups under Bonferroni correction

***p < .001

For girls and women, a significant overall difference was also found, with *F* (3, 1583) = 15.70 (*p* < 0.001). Post-hoc tests with Bonferroni corrections α (i.e, the corrected α = 0.008) were further conducted. There was no difference between older adult women (*M* = 4.10, *SD* = 0.86) and young adult women (*M* = 4.13, *SD* = 0.57), with *d* = 0.05, *p* = 0.970, a small effect size. However, young adult women (*M* = 4.13, *SD* = 0.57) had higher levels of functionality appreciation than both middle school (*M* = 3.77, *SD* = 0.84; *d* = 0.50, *p* < 0.001, a medium effect size) and high school (*M* = 3.94, *SD* = 0.67; *d* = 0.31, *p* < 0.001, a small effect size) girls. Moreover, high school girls also had higher levels of functionality appreciation than middle school girls (*d* = 0.22, *p* = 0.002; a small effect size). Finally, it should be noted that middle school girls had the lowest levels of functionality appreciation when compared to the other three female samples.

## Discussion

The current study examined the psychometric properties of the C-FAS in four age groups, inducing middle school students, high school students, young adults, and older adults. To the knowledge of the authors, the current work was the first study providing comprehensive psychometric evidence of the FAS in China, as well as the first to test age invariance and differences of the FAS. Our results showed that the C-FAS had sound psychometric properties in all age groups, and it was both gender invariant (boys and men vs. girls and women) and age invariant (adolescents vs. young adults vs. older adults) among samples from China. Moreover, significant age differences were also revealed.

In all age groups, the one-dimensional structure of the FAS [2] was successfully replicated. Moreover, the factor loadings of the C-FAS were large in all age groups, and this was also true for all sub-samples. Thus, the one-dimensional structure of the FAS was robust in the Chinese context. The Cronbach’s $\alpha$ values for the C-FAS were high in all age groups, suggesting the scores of C-FAS showed good internal consistency reliability which is in line with previous validation studies [2, 7–9]. In the current work, as hypothesized, the scores of C-FAS were found to be significantly and positively related to positive psychological constructs (e.g., body appreciation and self-compassion) but significantly and negatively related to negative psychological constructs (e.g., body dissatisfaction and eating disorder symptomatology). These correlational findings provide evidence for the construct validity of the C-FAS in all age groups in the Chinese context.

Interestingly, the association between functionality appreciation and BMI was only significant in older adults. Body functionality refers to what the body can do or is capable of doing [2]. Unlike adolescents and young adults who are younger and more energetic, older adults are more vulnerable to the influence of negative health conditions (e.g., overweight/obesity [76, 77] and disability [78]. Furthermore, previous research suggests that higher BMI in older adults was significantly related to poor physical functioning, including daily physical activities (e.g., lifting/carrying groceries, and walking a few blocks) [77, 79]. Thus, as higher BMI is more likely to negatively affect older adults’ physical functioning, older adults with higher BMI may also be less likely to appreciate what their bodies are capable of doing.

Regarding the association between functionality appreciation and body image inflexibility, small and negligible associations were identified among adolescents and young adults, but medium and significant associations were identified among older adults. Body image inflexibility is conceptualized as an unwillingness to experience negative thoughts and emotions about body weight and appearance [80]. Due to aging, older adults experience decreased physical functioning, which may make them more sensitive to the influence of body weight and appearance on physical functioning. Indeed, among older adults who endorsed higher functionality appreciation, body image inflexibility appeared to be less of an issue which, relative to their younger counterparts, may reflect a greater reliance of and value for physical wellbeing in older adults.

In line with previous validation studies [2, 7–9], the C-FAS was found to be invariant across gender in all age groups, indicating that it is relatively safe to make gender comparisons on functionality appreciation with the C-FAS in different Chinese age groups. Moreover, while we found that there were no gender differences in adolescents and young adults, there were small gender differences in older adults. According to a study investigating body image in men and women over the life span [81], older age was significantly associated with lower perceived importance of physical appearance in men, but not women. Thus, the finding of a slightly higher functionality appreciation in older men than older women may reflect potential gender differences in aging regarding the importance of physical appearance. Furthermore, unlike the common findings of males having a higher level of body appreciation than females on body appreciation [82], gender differences in functionality appreciation may be trivial, with nonsignificant gender differences commonly reported [2, 7–9]. This might be due to core differences between functionality appreciation and body appreciation. Functionality appreciation is focusing on the appreciation of body functioning which is more related to biological factors (e.g., physical function of the bodies); however, body appreciation is about the appreciation of physical appearance which is more related to social factors (e.g., societal body ideals). From the biological perspective, there are limited sex differences in physical functioning [83]; but, from the social perspective, women receive more daily appearance pressure from many sources including the media [84] which may make them less likely to appreciate their appearances than men.

Finally, the C-FAS was found to be invariant across age groups, indicating that it is reliable to make age comparisons on functionality appreciation by using the C-FAS. Moreover, we found significant overall age differences in functionality appreciation. The findings generally suggest that functionality appreciation increases with age for both boys and men as well as girls and women, even though certain age group comparisons were not statistically significant. With the aging process, humans may start to appreciate more about what their bodies can do, especially in older ages when body functionality greatly affects humans’ health-related quality of life [85]. These findings are also supported by positive body image theory and related constructs (e.g., body appreciation) which suggest that a shift in focus to health and functionality, and away from physical appearance, occurs with age in the United States context [37, 38, 86]. While continued research is needed in this domain, it may also be the case that this focus on health and functionality overlaps with other aspects of aging like interpersonal, occupational, and relational stability and success, all of which support one’s sense of self that is separated from aesthetic qualities (e.g., physical appearance) [37].Taken together, the current findings with age-related differences in functionality appreciation in the Chinese context, namely the observed higher levels of functionality appreciation in older Chinese adults, contribute to and build upon this conceptualization of positive body image.

The key strengths of the current study are the wide age ranges, from adolescents to older adults, as well as the examination of age invariance and differences of the FAS in an underrepresented cultural context. Strengths aside, the current work is not free from limitations. First, all samples in the current work were obtained by convenience sampling methods; thus, caution should be used in generalizing these findings to the general Chinese population or clinical populations (e.g., patients with eating disorders). Second, even though age invariance was met for the C-FAS, the participants were recruited from different locations and through different data collection methods (e.g., online survey in young adults vs. paper-and-pencil survey in adolescents and older adults) which should be considered in interpreting the current findings regarding significant age differences. Third, invariance tests by certain important factors, such as gender identity and sexual orientation [87, 88], were not conducted because we did not collect such information. Fourth, the test–retest reliability of the C-FAS for the four age groups was not assessed; thus, the temporal stability of the C-FAS remains unknown. Third, all data used in the current work were cross-sectional; thus, causal relationships between functionality appreciation as measured by the C-FAS and other constructs (e.g., eating disorder symptomatology) are not guaranteed. Future research on this topic should consider the limitations of the current work to advance the empirical body image literature.

## Conclusions

Overall, in the current work, the C-FAS was found to be a unidimensional measure that was invariant across gender and age. The C-FAS also showed sound psychometric properties for all samples of different ages, including middle school students, high school students, young adults, and older adults. To date, there has been limited and preliminary research related to functionality appreciation in China, and thus the validation of the C-FAS is a key first step in the service of generating research in these areas within the Chinese context. Specifically, future studies could adopt qualitative methods involving interviews and/or longitudinal methods to better clarify the relationships between functionality appreciation and aging as well as other related predictors and outcomes (e.g., BMI and eating behaviors).

## Data Availability

The data are available upon request from the corresponding author.

## Abbreviations

FAS: Functionality Appreciation Scale
EFA: Exploratory factor analyses
CFA: Confirmatory factor analyses
WEIRD: Western, Educated, Industrialized, Rich, and Democratic
C-FAS: Chinese version of the FAS
BAS-2: Body Appreciation Scale-2
EDI-BD: Body Dissatisfaction subscale of the Eating Disorder Inventory
BI-AAQ-5: The 5-item Body Image-Acceptance and Action Questionnaire
K6: The 6-item Kessler Psychological Distress Scale
EDE-QS: Eating Disorder Examination-Questionnaire
SCS-SF: Self-Compassion Scale—Short Form
BMI: Body Mass Index
WLSMV: Mean- and variance-adjusted weighted least squares estimator
MLR: Maximum likelihood with robust standard errors
CFI: Comparative Fit Index
TLI: Tucker–Lewis Index
RMSEA: Root Mean Square Error of Approximation
SRMR: Standardized Root Mean square Residual
MCAR: Missing Completely At Random

## Footnotes

1 The SCS-SF was not included to limit the number of items on this online survey and reduce the survey cost and burden for online participants.

2 We did not use the SCS-BF given the study design for the older adults’ project did not include this measure of self-compassion.
